# Sevoflurane Modulates AKT Isoforms in Triple Negative Breast Cancer Cells. An Experimental Study

**DOI:** 10.3390/cimb43010022

**Published:** 2021-06-02

**Authors:** Crina E. Tiron, Emilia Patrașcanu, Paula A. Postu, Irina C. Vacarean Trandafir, Adrian Tiron, Ioana Grigoras

**Affiliations:** 1 TRANSCEND Research Center, Regional Institute of Oncology, 700483 Iasi, Romania; transcendctiron@iroiasi.ro (C.E.T.); paula.postu@iroiasi.ro (P.A.P.); irina.trandafir@iroiasi.ro (I.C.V.T.); 2 Department of Anaesthesia and Intensive Care, School of Medicine, “Grigore T. Popa” University of Medicine and Pharmacy, 700115 Iasi, Romania; patrascanu.emilia@umfiasi.ro (E.P.); ioana.grigoras@umfiasi.ro (I.G.); 3 Department of Anesthesia and Intensive Care, Regional Institute of Oncology, 700483 Iasi, Romania

**Keywords:** AKT, breast cancer, EMT, HIF, sevoflurane, vimentin

## Abstract

(1) Background: Triple negative breast cancer (TNBC) is a highly aggressive tumor, associated with high rates of early distant recurrence and short survival times, and treatment may require surgery, and thus anesthesia. The effects of anesthetic drugs on cancer progression are under scrutiny, but published data are controversial, and the involved mechanisms unclear. Anesthetic agents have been shown to modulate several molecular cascades, including PI3K/AKT/mTOR. AKT isoforms are frequently amplified in various malignant tumors and associated with malignant cell survival, proliferation and invasion. Their activation is often observed in human cancers and is associated with decreased survival rate. Certain anesthetics are known to affect hypoxia cell signaling mechanisms by upregulating hypoxia-inducible factors (HIFs). (2) Methods: MCF-10A and MDA-MB 231 cells were cultivated and CellTiter-Blue^®^ Cell Viability assay, 2D and 3D matrigel assay, immunofluorescence assays and gene expressions assay were performed after exposure to different sevoflurane concentrations. (3) Results: Sevoflurane exposure of TNBC cells results in morphological and behavioral changes. Sevoflurane differently influences the AKT isoforms expression in a time-dependent manner, with an important early AKT3 upregulation. The most significant effects occur at 72 h after 2 mM sevoflurane treatment and consist in increased viability, proliferation and aggressiveness and increased vimentin and HIF expression. (4) Conclusions: Sevoflurane exposure during surgery may contribute to cancer recurrence via AKT3 induced epithelial–mesenchymal transition (EMT) and by all three AKT isoforms enhanced cancer cell survival and proliferation.

## 1. Introduction

Multiple factors, both biological and therapy-related, influence cancer evolution and progression. Despite significant advances in oncological therapies, cancer remains a major cause of mortality. Tumor metastasis, a complex process that requires release of metastatic cells from a primary tumor, invasion and (local, lymphatic, intravascular) migration and subsequent extravasation and proliferation in target tissues, is accounted for 90% of cancer death [1,2,3]. Cellular and molecular events associated with the metastatic process may be significantly influenced by many factors during and immediately after surgery [4,5]. Surgical resection is a widely used treatment for solid tumors, including breast cancer. Various studies suggest that the choice of anesthesia technique/drugs could affect long-term outcome after surgery [6,7,8]. Deegan et al. demonstrated that serum of patients with breast cancer surgery, taking place under two different anesthesia techniques, induce different effects on cancer cell proliferation and migration, thus differently influencing cancer recurrence/metastasis [4]. Inhalational anesthetics and opiates analgesics have been associated with impaired immunity and increased tumor recurrence [9,10,11,12].

Certain anesthetics (e.g., isoflurane, sevoflurane, halothane) are known to affect hypoxia cell signaling mechanisms by up-regulating HIFs. HIFs are also implicated in tumorigenesis and metastasis influencing angiogenesis, energy metabolism, cell proliferation, apoptosis, and cell migration [13,14]. Other anesthetics (e.g., propofol and etomidate) are able to stimulate the EMT initiation through the increment of mesenchymal markers expression [15]. Volatile anesthetics (e.g., halothane, isoflurane, sevoflurane) have been implicated in angiogenesis and immunosuppression, induced apoptosis of immune competent cells (NK cells and T-lymphocytes), but also have a promoting effect on tumor metastasis [10,11,12,13,14,15,16]. Although sevoflurane is nowadays the most frequently used inhaled anesthetic, its direct influence on cancerous cells is mainly unknown.

Anesthetic agents (e.g., isoflurane, sevoflurane) have been shown to modulate several molecular cascades in various tissues, including PI3K/AKT/mTOR [17,18]. AKT, also known as protein kinase B (PKB), plays a key role in signaling downstream of growth factors and other stimuli, regulating critical cellular functions, including proliferation and survival. AKT abnormal hyperactivation by gene amplification or somatic mutation is frequently associated with human pathology, including cancer [19,20].

In humans, there are three AKT isoforms (AKT1, AKT2 and AKT3) containing the following four domains: PH domain, linker region, catalytic domain and C-terminal regulatory region. Most studies using individual knockout of those three isoforms suggested non-redundant functions of AKT proteins [20,21,22,23,24,25,26,27,28]. The mechanisms by which those isoform signaling can be conveyed are different: post-translational modifications, regulation of kinase activity, substrate specificity and subcellular localization [29,30,31,32].

In normal tissues AKT1 has a wide tissue distribution; AKT2 is highly expressed in adipocytes and muscle, whereas AKT3 is mainly expressed in the testes, lungs and brain [22,25,26,27,28,29,30,31,32,33,34,35].

In cancer, AKT1 has been found to be overexpressed in human gastric, breast and ovarian cancers; AKT2 was amplified and overexpressed in ovarian, pancreatic, hepatic, colorectal cancer and glioma, while AKT3 has been reported in progression of breast, prostate, ovarian cancer, and malignant melanoma [36,37,38,39]. Several studies have suggested that AKT family members serve distinct roles in invasion, migration and metastatic dissemination [40,41,42,43].

Therefore, in order to investigate sevoflurane effects on tumor progression and metastasis, we performed an experimental study aiming to evaluate the effect of sevoflurane on AKT isoforms expression in human breast cancer cells and its effects on viability, proliferation, aggressivity and EMT.

## 2. Results

### 2.1. Sevoflurane Exposure Modulates Cells Viability and Proliferation Rate

**Figure 1:**
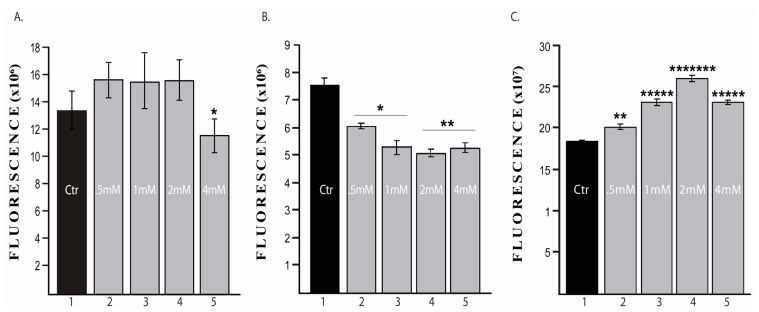
Cell viability. (**A**) Normal mammary epithelial cells MCF10a; (**B**) human breast adenocarcinoma cell line MDA-MB 231 at 24 h; (**C**) human breast adenocarcinoma cell line MDA-MB 231 at 72 h; (1. Untreated; 2–5 treated with 0.5 mM, 1 mM, 2 mM, and 4 mM sevoflurane), * *p* < 0.05; ** *p* < 0.005; ***** *p* < 0.000005; ******* *p* < 0.00000005. *n* = 3 (8 wells per group).

We assessed sevoflurane effects on cell viability and cell proliferation rate of triple negative breast cancer cell line MDA-MB-231 and normal breast cell line MCF-10A in 2D culture system. Viability of normal mammary epithelial cells (MCF-10A) was unaffected by 0.5–2 mM sevoflurane concentrations at 72 h (Figure 1A), while 4mM concentration significantly reduced number and altered cell phenotype. In human breast adenocarcinoma cell line (MDA-MB-231) viability was dynamic, significantly decreasing 24 h post sevoflurane exposure (Figure 1B) and significantly increasing at 72 h post sevoflurane treatment (Figure 1C), results observed at all concentrations. The most significant changes have been observed in the cells treated with 2 mM sevoflurane concentration (Figure 1(B, column 4 and C, column 4)).

**Figure 2:**
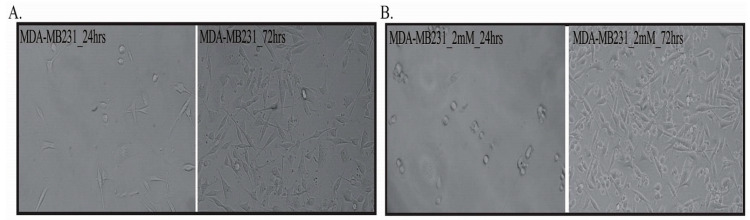
Morphological aspects of MDA-MB 231 cells. (**A**) Untreated; (**B**) treated. *n* = 2; 10×.

Moreover, the sevoflurane exposed MDA-MB-231 cells acquired an uncharacteristic round shape at 24 h, and they recovered their mesenchymal-like phenotype at 72 h time point (Figure 2, Supplementary Figure S1).

These data indicate that different sevoflurane concentrations reduced proliferation rate of breast cancer cells and their morphological phenotype dramatically changed.

### 2.2. Sevoflurane Exposure Increases Aggressivity

**Figure 3:**
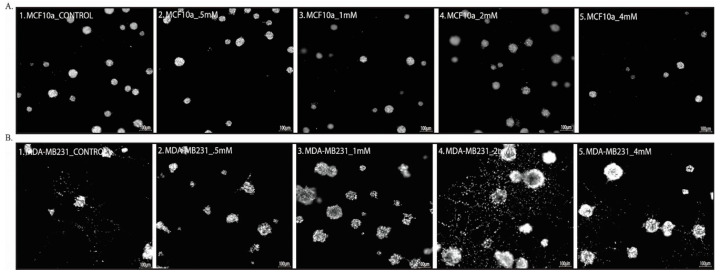
Morphological aspects of 3D matrigel culture. (**A**) Normal mammary epithelial cells MCF10A; (**B**) human breast adenocarcinoma cell line MDA-MB231; (1. Untreated; 2–5 treated with 0.5 mM, 1 mM, 2 mM, and 4 mM sevoflurane). *n* = 2.

As there is a correlation between sevoflurane concentration and cancer cell proliferation in 2D system, we evaluated the effect of sevoflurane in a 3D matrigel model. Our data demonstrate that, with increasing sevoflurane concentration, the breast carcinoma cell line (MDA-MB-231) forms large colonies with a higher number of invasive cell projection, particularly at 2mM (Figure 3), indicating a migratory behavior with increased aggressiveness, as shown by Gjerdrum [44].

On the contrary, the sevoflurane exposed normal mammary cells (MCF10A) maintained the same spheroidal phenotype as the untreated MCF10A cells (Figure 3A), while the number of spheroids was reduced by 4 mM sevoflurane administration (Figure 3(A_5)).

Together, these findings suggest that high sevoflurane level amplify the mesenchymal-like invasiveness of metastatic breast carcinoma cells.

### 2.3. Sevoflurane Exposure Affects AKT Isoforms Expression

We assessed the influence of 2 mM sevoflurane concentrations on AKT isoforms expression. This concentration was chosen based on the above mentioned results, showing that cancer cells presented the most robust proliferation changes at 2 mM sevoflurane concentration.

**Figure 4:**
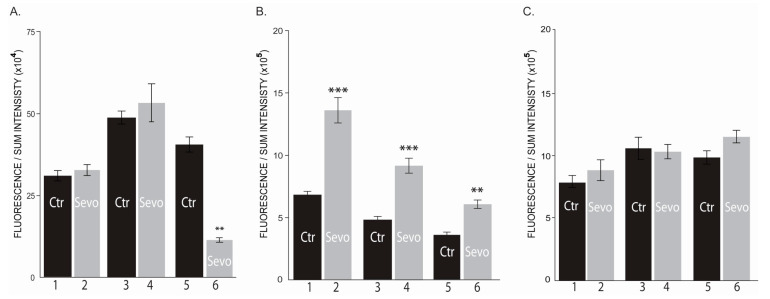
Level of AKT isoforms in MCF-10A. (**A**) AKT1 expression level; (**B**) AKT2 expression level; (**C**) AKT3 expression level (1, 2–6 h; 3, 4–24 h; 5, 6–72 h; Ctr-untreated cells; sevo-treated cells with 2 mM sevoflurane; h-hours). ** *p* < 0.005; *** *p* < 0.0005. *n* = 2.

In normal breast cells sevoflurane exposure has different impact on the three AKT isoforms expression. AKT1 level was significantly reduced at 72 h (Figure 4A), Supplementary Figure S2A), AKT2 was significantly upregulated by sevoflurane treatment at all timepoints (Figure 4B), Supplementary Figure S3A), while AKT3 expression was not affected (Figure 4C, Supplementary Figure S4A).

**Figure 5:**
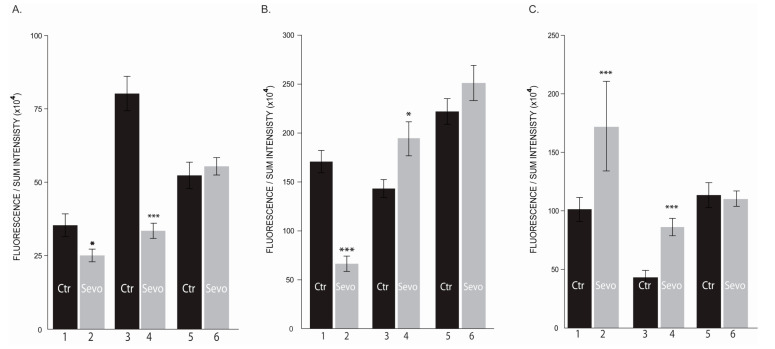
Level of AKT isoforms in MDA-MB231. (**A**) AKT1 expression level; (**B**) AKT2 expression level; (**C**) AKT3 expression level (1, 2–6 h; 3, 4–24 h; 5, 6–72 h; Ctr-untreated cells; sevo-treated cells with 2 mM sevoflurane; h-hours) * *p* < 0.05; *** *p* < 0.0005. *n* = 2.

In the triple negative breast cancer cells, sevoflurane exposure dynamically adjusts the AKT isoforms expression (Figure 5A–C, Supplementary Figures S2B–S4B).

At the end of 6 h treatment, a significant drop in AKT1 and AKT2 expressions was observed (Figure 5). On the other hand, at the same time point, a major upregulation in AKT3 isoform expression was noticed (Figure 5(C_2)). Later, at 24 h, AKT1 expression was further downregulated (Figure 5(A_4)) and the AKT3 expression was further upregulated (Figure 5(C_4)), the AKT2 shifted its expression pattern being significantly increased (Figure 5(B_4)). Although the expression levels vary greatly between AKT isoforms, sevoflurane showed no influence at 72 h on MDA-MB-231 cells, sevoflurane exposed and unexposed cells presenting a similar expression of AKT1, AKT2 or AKT3 isoforms (Figure 5). Altogether, these results suggest that the sevoflurane exposure highly influences the AKT isoforms expression in the first 24 h.

### 2.4. Sevoflurane Differently Regulates Intermediate Filaments (IFs) Proteins Levels

**Figure 6:**
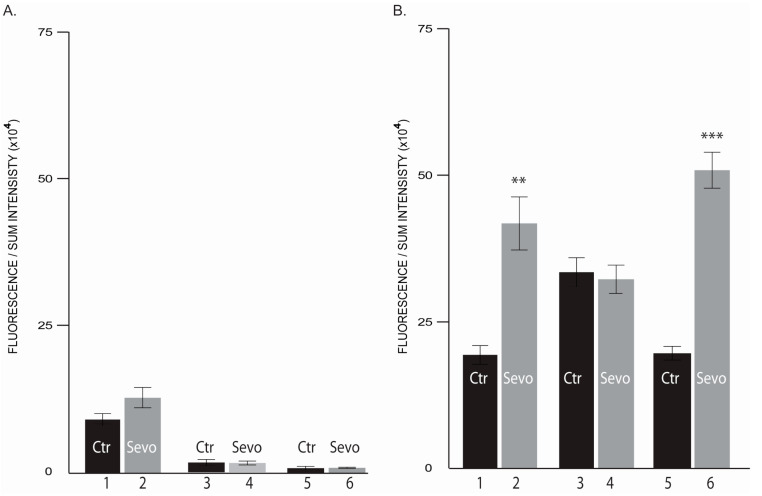
Level of Vimentin. (**A**) Normal mammary cells MCF-10A; (**B**) human breast adenocarcinoma cell line MDA-MB231 (1, 2–6 h; 3, 4–24 h; 5, 6–72 h; Ctr-untreated cells; Sevo-treated cells with 2 mM sevoflurane; h-hours). ** *p* < 0.005; *** *p* < 0.0005. *n* = 2.

We demonstrate that sevoflurane treatment modulates vimentin, a mesenchymal-specific IFs protein, expression in triple negative breast cancer cells. Sevoflurane exposed versus unexposed MDA-MB-231 cells show at 6 h a significant increase in vimentin expression (Figure 6, Supplementary Figure S5B) and then, at 24 h, vimentin expression dropped, having a similar expression level as untreated MDA-MB-231 cells (Figure 6, Supplementary Figure S5B).

Surprisingly, at 72 h, a significant vimentin expression burst was detected in MDA-MB-231 cells (Figure 6, Supplementary Figure S5B).

Regarding non-malignant MCF-10A breast cells, the vimentin expression presented a decreasing trend over the 72 h experiment despite sevoflurane administration (Figure 6A, Supplementary Figure S5A).

The significantly increased levels of Vimentin protein expression at 72 h demonstrates that sevoflurane exposure induce EMT via AKT3 isoform.

### 2.5. Sevoflurane Exposure Modulates HIF-1α Expression

**Figure 7:**
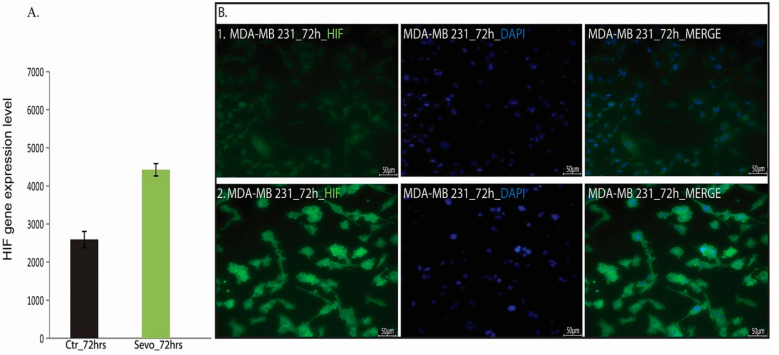
HIF-1α expression. (**A**) HIF-1α gene expression level; (**B**) HIF-1α protein expression level; (1. 6 h post-sevoflurane exposure; 2. 72 h post-sevoflurane exposure). *n* = 1 (**A**), *n* = 2 (**B**).

In our experimental setup we found an increased HIF-1α genes (Figure 7A) and protein expression (Figure 7B) at 72 h after sevoflurane exposure in cancer cell lines.

## 3. Discussion

Inhaled anesthetics are frequently used in different types of surgery, including oncological surgery, despite emerging evidence of potential deleterious effects. Although sevoflurane is the mostly used volatile agent due to its pharmacokinetic and pharmacodynamic advantages, little is known about its influence on cancer cells. Identifying the effects of the inhaled anesthetics on tumor cells will improve understanding of tumor recurrence/metastasis after surgery and may lead to changes in clinical practice. In this study, we investigated the effects of sevoflurane on triple negative breast cell line and some of the potential mechanisms involved.

Treating triple negative breast cancer, a subtype of breast cancer that lacks the receptors for estrogen, progesterone and HER2 (human epidermal growth factor receptor 2), remains challenging although great progresses were done over the recent years [45]. Being either lumpectomy or mastectomy, oncosurgery remains an effective treatment for primary breast tumor removal [46], so anesthesia is unavoidable. The Ecimovic et al. in vitro study showed that sevoflurane affects human triple positive and triple negative breast cancer cells, suggesting that volatile anesthetics may induce tumorigenic effects [47]. Similarly, we demonstrated that sevoflurane promoted the proliferation of the MDA-MB-231 cells. Moreover, we showed that sevoflurane enhanced the aggressive behavior of triple negative breast cancer cells in 3D matrigel assay. Other studies reported that in certain tumor cells (lung, pancreas, colon [48,49,50]) sevoflurane inhibited tumor growth and those results could be explained by differences in to cell type, time of exposure, concentration, etc. Our data demonstrate that initially (24 h post sevoflurane exposure) sevoflurane indeed inhibited cancer cell proliferation, while at 72 h there is a significant increase of proliferation and changes in morphological phenotype. These phenomena did not occur in normal breast cell line, except 4mM concentration. We speculate that high sevoflurane concentration (2 mM) induces EMT and concomitantly increased activity of the cancer stem cell population, which in the end will promote invasiveness and cancer aggressiveness.

To elucidate the underlying mechanism, we examined the effects of sevoflurane on AKT isoforms, which were reported to be involved in major cellular processes [17,18,19,20,21,22,23,24,25,26,27,28]. The overexpression of AKT isoforms in different types of malignancies is well documented [51]. While different studies revealed different results regarding the involvement of AKT1 and AKT2 in mammary carcinomas, AKT3 dysfunctions are often correlated with triple negative breast cancer [52]. Our study demonstrates a time-dependent expression of AKT1,2,3 in MDA-MB 231 cells after 2mM sevoflurane exposure. It has been reported that AKT1 does not promote invasive phenotype, while AKT3 is required for TNBC proliferation and tumor growth [43]. Congruent with this observation, we found a significant drop in AKT1 at 6 and 24 h post 2 mM sevoflurane exposure, and only AKT3 isoform was significantly upregulated at those time points.

Recent studies showed that intermediate filaments (IFs) are involved in signaling pathways that regulate cell growth, resistance to apoptosis and motility. Vimentin, a mesenchymal-specific IFs protein, is a distinct feature of EMT [53]. The EMT activation is correlated with molecular changes as shifts in cadherins expression [54], cytokeratin loss, vimentin and collagen increased expressions [53,55]. Moreover, PI3K-AKT pathway is essential to maintain the CSC-like phenotype and EMT characteristics in breast cancer cells [56]. Sevoflurane significantly enhances EMT, as demonstrated by upregulation of its commonly used marker, vimentin, at 6 and 72 h post sevoflurane exposure. This further supports the AKT3 key role in sevoflurane induced tumor growth, invasion and tumor recurrence.

The volatile anesthetics upregulate HIFs [10,11,12,13,14,15,16] and HIF overexpression is associated with an unfavorable prognosis in breast cancer [57,58]. HIF-1α has been found to promote EMT in many human malignancies leading to increased cancer aggressiveness [59]. Additionally, PI3K/AKT and the MAP kinase Erk1/2, have been implicated in upregulating of HIF-1α [17]. Here, we demonstrate that 2 mM sevoflurane exposure increases HIF-1α genes and protein expression. In our design, HIF-1α further validates that sevoflurane exposure induces EMT.

Our in vitro study investigates sevoflurane effects on breast normal and cancer cells and some of the involved mechanisms. Our results open the way to further research, which should investigate in vivo the cumulative effects of anesthetics, surgery and related factors, but also the interplay of effects on cancer cells and on immune system.

## 4. Materials and Methods

We designed an in vitro experiment using 2D and 3D cultures of two breast cell lines (a human cancer cell line and human normal breast cell line) exposed to different sevoflurane concentrations.

We used a human breast adenocarcinoma cell line—MDA-MB-231 (ATCC^®^, Rockville, MD, USA, ATCC), a triple negative aggressive form of breast cancer, and normal human mammary epithelial cells—MCF-10A (ATCC^®^ Rockville, MD, USA, ATCC). Cell lines are a generous gift from James Lorens (Bergen Bio, Bergen, Norway).

All cell cultures, both cancer and normal cells, were divided in 5 groups handled in the same manner: without sevoflurare exposure (G1) and with sevoflurane exposure—0.5 mM (G2), 1 mM (G3), 2 mM (G4) and 4 mM (G5). All 2D groups were assessed at the end of 6 h exposure, and 24, and 72 h after exposure for AKT expression, cell viability and proliferation, EMT by vimentin expression and HIF-1α expression. All 3D groups were assessed for cell phenotype.

### 4.1. Reagents

Horse serum (Sigma, St. Luis, MO, USA ), EGF (Sigma, St. Luis, MO, USA), insulin (Sigma, St. Luis, MO, USA), hydrocortisone (Sigma, St. Luis, MO, USA), cholera toxin (Sigma, St. Luis, MO, USA), Pen/Strep (Sigma, St. Luis, MO, USA), bovine serum (Sigma-Aldrich, St. Luis, MO, USA), CellTiter-Blue^®^ Cell Viability Assay (Promega, Madison, WI, USA), Matrigel (Cornning, Corning, NY, USA), rabbit polyclonal anti-human AKT1 antibody (Sigma Aldrich, SAB3500216, St. Luis, MO, USA), rabbit polyclonal anti-human AKT2 antibody (Sigma Aldrich, SAB4300423, St. Luis, MO, USA), rabbit monoclonal anti-human AKT3 antibody (Cell Signaling Technology, E1Z3W, Leiden, Netherlands), rabbit monoclonal anti-human Vimentin antibody (Cell Signaling Technology, D21H3, Leiden, Netherlands), rabbit monoclonal anti-human Hif-1α antibody (Abcam, 179483, Cambridge, UK), fluorescein goat anti-rabbit IgG (ThermoFisher Scientific, F2765, Eugene, OR, USA), Prolong (R) Gold antifade with DAPI (Cell Signaling Technology, 8961S, Leiden, Netherlands), 4% paraformaldehyde (Sigma Aldrich, St. Luis, MO, USA), PBS (Santa Cruz, Heidelberg, Germany), saponin (Merck, Kenilworth, NJ, USA), Triton X 100 (Sigma Aldrich, St. Luis, MO, USA), digitonin (Promega, Madison, WI, USA), goat serum (Dako, Denmark, Europe), Fetal bovine serum (Sigma, St. Luis, MO, USA), TRIzol (Invitrogen, Carlsbad, CA, USA), First Strand cDNA Synthesis Kit (Invitrogen, Carlsbad, CA, USA), and Power SYBR Green PCR Master Mix (Applied Biosystems™, Foster City, CA, USA).

### 4.2. Cell Culture

MCF-10A cells were cultured in DMEM/F12 supplemented with 5% horse serum, 20 ng/mL EGF, 10 ug/mL insulin, 0.5 ng/mL hydrocortisone, 10 ng/mL cholera toxin, 1% Pen/Strep. MDA-MB-231 cells were cultured in F-12K Medium, supplemented with 100 U/mL of penicillin and 100 μg/mL of streptomycin and 5% bovine serum.

### 4.3. Cell Viability and Sevoflurane Exposure

Cells were seeded into a 96-well flat bottom tissue culture plate at a density of 2000 cells/well and allowed to adhere to the plate by incubating at 37 °C under 5% CO_2_ overnight. Following cell attachment, the cells were incubated for 6h with a sevoflurane solution having one of the following concentrations: 0.5, 1, 2 and 4 mM. 24 h and 72 h post sevoflurane exposure, 50 μL of cell viability solution (CellTiter-Blue^®^ Cell Viability Assay, Promega, Madison, WI, USA) was added to each well and the plate was re-incubated for 4 h before microplate fluorescence recording (FilterMax F5, Sunnyvale, CA, USA).

### 4.4. The 3D Matrigel Assays

1000 cells were seeded in Ibidi plates, between 2 layers of Matrigel (BD Matrigel Matrix, Growth Factor Reduced (BD Biosciences, San Jose, CA, USA)) and cultured for 14 days before microscopy analysis (TissueGnostic rig, Vienna, Austria). Twelve hours post seeding, 3D embedded cells were treated with a sevoflurane solution at concentrations either 0.5, 1, 2 or 4 mM for 6 h. After 6h, the sevoflurane solution was removed and replaced with normal 3D matrigel medium (medium corresponding to every cell type supplemented with 2% FBS and 1% matrigel).

### 4.5. Immunofluorescence

Several 96-well flat bottom culture plates were seeded with MCF-10A (one half of the plate) and MDA-MB-231 cells (the other half of the plate) in 2D culture system at 37 °C under 5% CO_2_, each well containing 2000 cells. One plate was used as control and was not exposed to sevoflurane.

After 24 h, plates containing attached cells were treated by incubation with 2 mM sevoflurane solution for 6 h, and each plate was fixed with 4% paraformaldehyde at a different time points: either immediately after sevoflurane treatment, at 24 h or at 72 h post sevoflurane exposure. For each corresponding fixation point, we fixed MCF-10A and MDA-MB-231 cells from parallel 2D untreated cultures (unexposed plate). After cell fixation, the plates were washed with PBS for 5 min at room temperature. For immunofluorescence staining the cell membranes were permeabilized for 2 h at room temperature using a permeabilization solution containing 0.3% saponin, 0.3% Triton X 100 and 0.3% digitonin. Diluted primary antibodies, anti-AKT1 (1:25), anti-AKT2 (1:25), anti-AKT3 (1:25), anti-vimentin (1:15) and anti-HIF-1α (1:25) were incubated for 72 h at 4° C. These antibodies were diluted using an antibody dilution solution containing 0.3% saponin, 0.3% Triton X 100 and 0.5% goat serum. The cells were washed twice with PBS (each washing step lasted for 5 min) and incubated with fluorescein goat anti-rabbit IgG (1:200) for 24 h at 4 °C (the antibody was diluted using an antibody dilution solution containing 0.3% saponin, 0.3% Triton X 100 and 2% fetal bovine serum). After fluorescein goat anti-rabbit IgG removal, cells were washed twice with PBS (each washing step had a 5 min duration) and the fluorescent dye was protected by incubating over night at 4 °C with Prolong (R) Gold antifade with DAPI. Pictures were acquired at 20× with Zeiss Axio Observer Z1 Microscope TissueGnostic using Tissue FAXS 4.2 software (Vienna, Austria). TissueQuest 6.0 (Vienna, Austria) software was used for quantitative cell analysis and to quantify sum intensity of florescence signal for each event.

### 4.6. Quantitative RT-PCR (qRT-PCR) Analysis

Molecular biology techniques were used to determine changes in HIF-1α expression in MDA-MB-231 cells, by assessing gene expression for specific molecular targets.

Total RNA extraction from 10^7^ cells was performed using TRIzol. The cells were lysed, and RNA purification was performed according to the manufacturer’s protocol. Total RNA concentration was measured by spectrophotometry at 260 nm and the RNA was stored at −20 °C until reverse transcription in cDNA was performed. cDNA was synthesized using the First Strand cDNA Synthesis Kit using the Corbett CGI-96 Palm-Cycler Thermal Cycler equipment. The complementary DNA obtained was used immediately or stored for a short time at −20 °C or for a longer term at −80 °C.

Quantitative Real-time PCR amplification was performed on a LightCycler 480 II (Roche) using the following primer pairs for HIF-1α: Fw: 5′-CACTACCACTGCCACCACTG-3′ and Rev: 5′-CCTTTTCCTGCTCTGTTTGG-3′. For the reaction, 5 μL of cDNA were incubated with 12.5 μL of Power SYBR Green PCR Master Mix and 1 μL of forward and reverse primers in a final volume of 25 μL. The primer melting temperature was 60° and the analysis was performed in triplicate. The obtained amplicons of different concentrations through RT-PCR amplification were compared to the concentration of ABL reference gene. The results were reported as percentages of increase/decrease of the gene expression compared to the control, as compared to the reference gene (ABL): % gene = number of amplicon genes/µL/reference gene (ABL) × 100.

### 4.7. Statistical Analysis

Statistical analysis was performed with GraphPad Prism 7, *t*-test. Data analysis results are presented as the mean ± S.E.M. Significance was established when *p* < 0.05.

## 5. Conclusions

Our study demonstrates that in vitro sevoflurane exposure of triple negative breast cancer cells results in morphological and behavioral changes. Sevoflurane differently influences the AKT isoforms expression in a time-dependent manner, with an important early AKT3 upregulation. The most significant effects occur at 72 h after 2 mM sevoflurane treatment and consist in increased viability, proliferation and aggressiveness and increased vimentin and HIF expression. Thus, we may conclude that sevoflurane exposure during surgery may contribute to cancer recurrence via AKT3 induced EMT and by all three AKT isoforms enhanced cancer cell survival and proliferation.

## Data Availability

The data will be publicly available by open access.

## Supplementary Materials

Supplementary materials are available online at https://www.mdpi.com/article/10.3390/cimb43010022/s1.
