# Supplementary material for: Sevoflurane Modulates AKT Isoforms in Triple Negative Breast Cancer Cells. An Experimental Study
Source: Curr Issues Mol Biol. 2021 Jun 2;43(1):264–75. doi: 10.3390/cimb43010022 (PMC8929147; doi:10.3390/cimb43010022)
Supplement: Supplementary file 1 [file cimb-43-00022-s001.zip › Information.pdf]

Figure S1: Morphological aspects of MDA-MB 231 cells. (**A**) 24 h; (**B**) 72 h; (1. Untreated; 2–5 treated with 0.5 mM, 1 mM, 2 mM, 4 mM);

Figure S2: Akt1 expression. (**A**) MCF-10a; (**B**) MDA-MB 231; (Control–untreated, sevoflurane–treated 2mM);

Figure S3: Akt2 expression. (**A**) MCF-10a; (**B**) MDA-MB 231; (Control–untreated, sevoflurane–treated 2mM);

Figure S4: Akt3 expression. (**A**) MCF-10A; (**B**) MDA-MB 231; (Control–untreated, sevoflurane–treated 2 mM);

Figure S5A: Vimentin expression. (**A**) MCF-10A; (Control–untreated, sevoflurane–treated 2 mM); Figure S5B: Vimentin expression. (**B**) MDA-MB 231; (Control–untreated, sevoflurane–treated 2 mM).
