# Supplementary figures and images for: Sevoflurane Modulates AKT Isoforms in Triple Negative Breast Cancer Cells. An Experimental Study
Source: Curr Issues Mol Biol. 2021 Jun 2;43(1):264–75. doi: 10.3390/cimb43010022 (PMC8929147; doi:10.3390/cimb43010022)

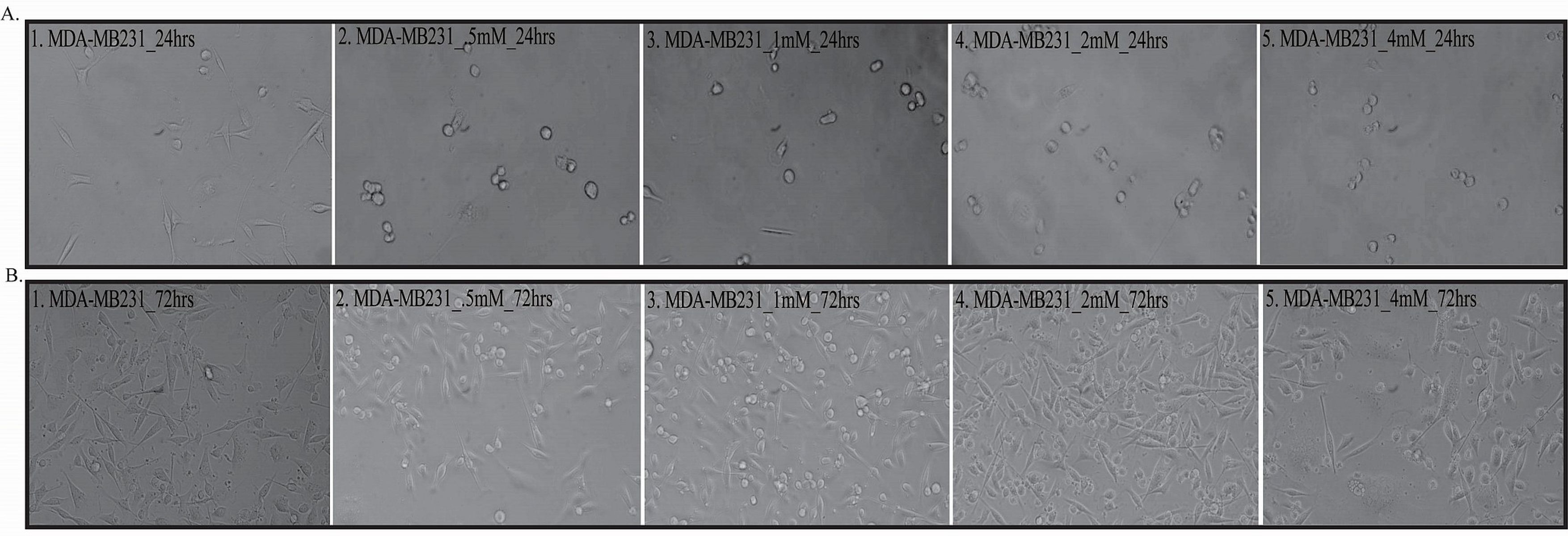

Supplement: Supplementary file 1 [file cimb-43-00022-s001.zip › SUPPLIMENTARY FIG. 1.jpg]

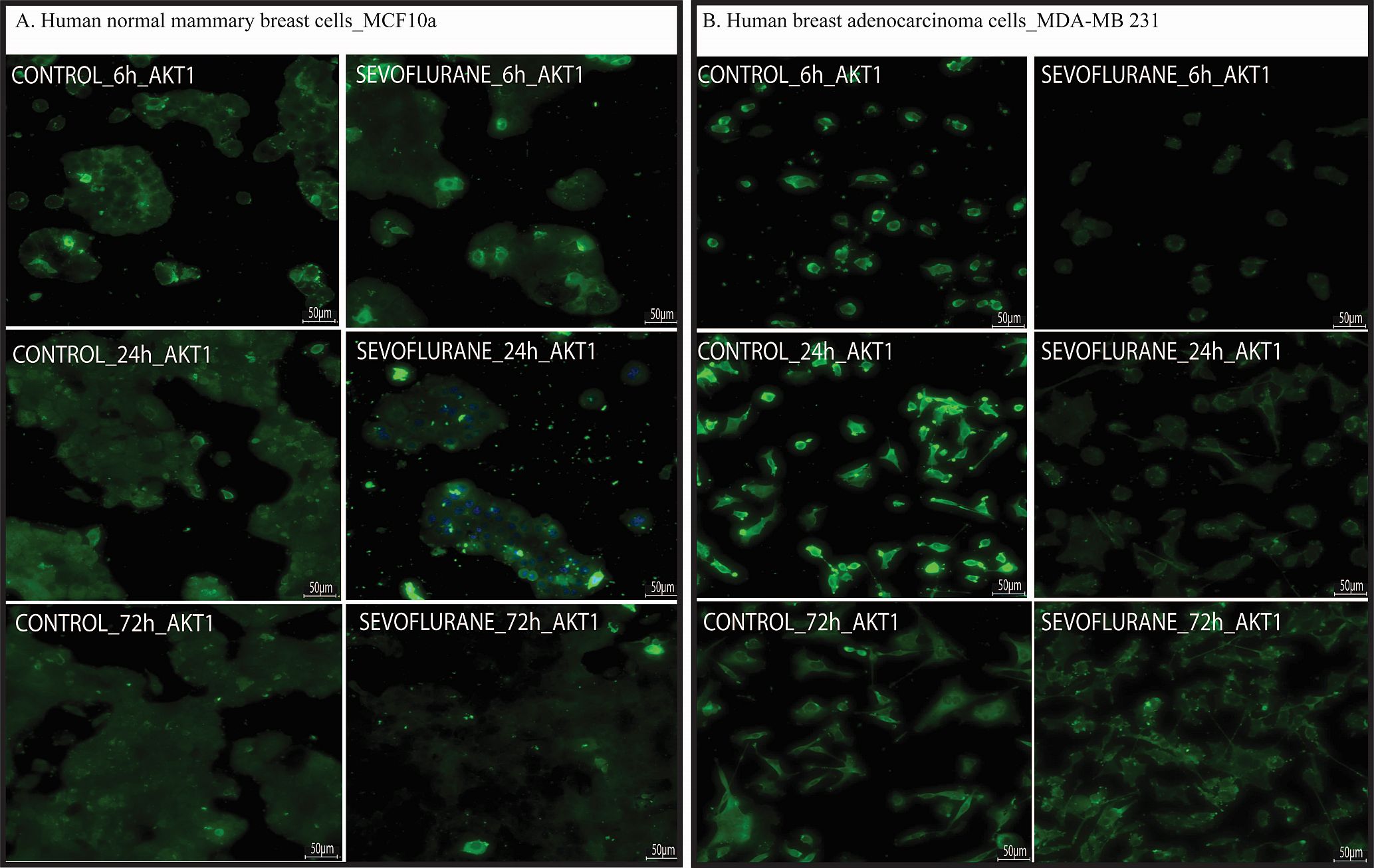

Supplement: Supplementary file 1 [file cimb-43-00022-s001.zip › SUPPLIMENTARY FIG. 2.jpg]

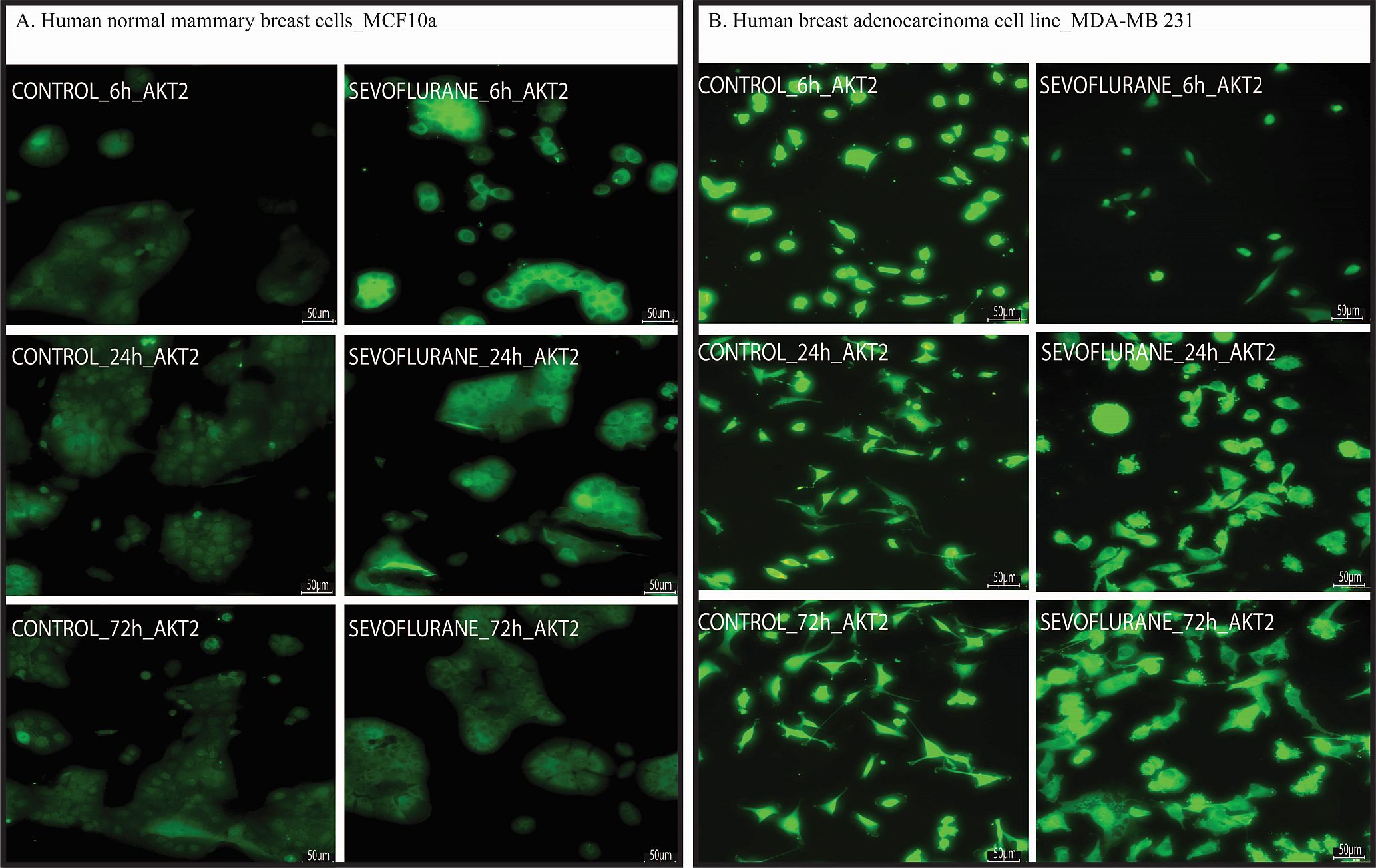

Supplement: Supplementary file 1 [file cimb-43-00022-s001.zip › SUPPLIMENTARY FIG. 3.jpg]

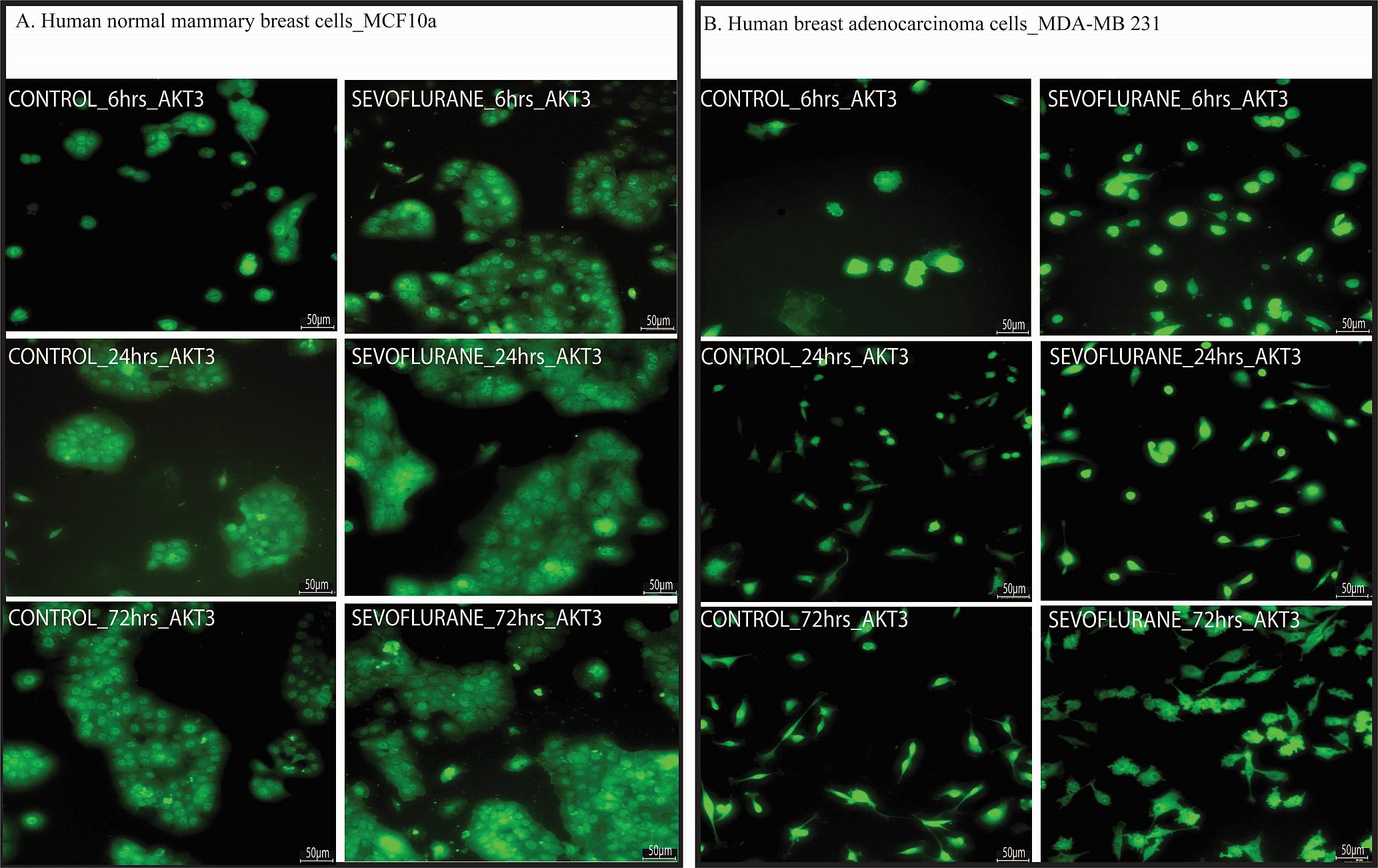

Supplement: Supplementary file 1 [file cimb-43-00022-s001.zip › SUPPLIMENTARY FIG. 4.jpg]

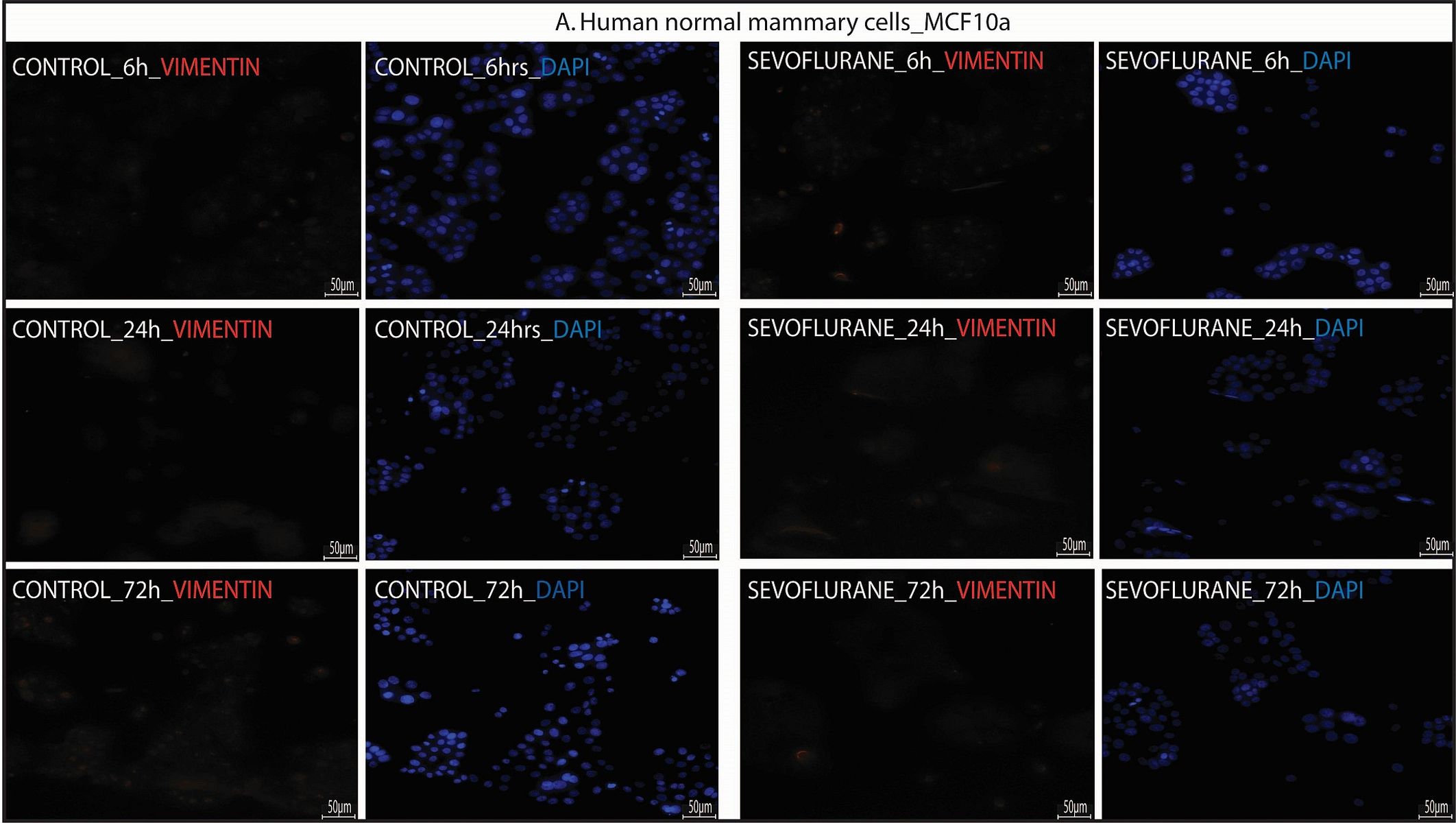

Supplement: Supplementary file 1 [file cimb-43-00022-s001.zip › SUPPLIMENTARY FIG. 5_A.jpg]

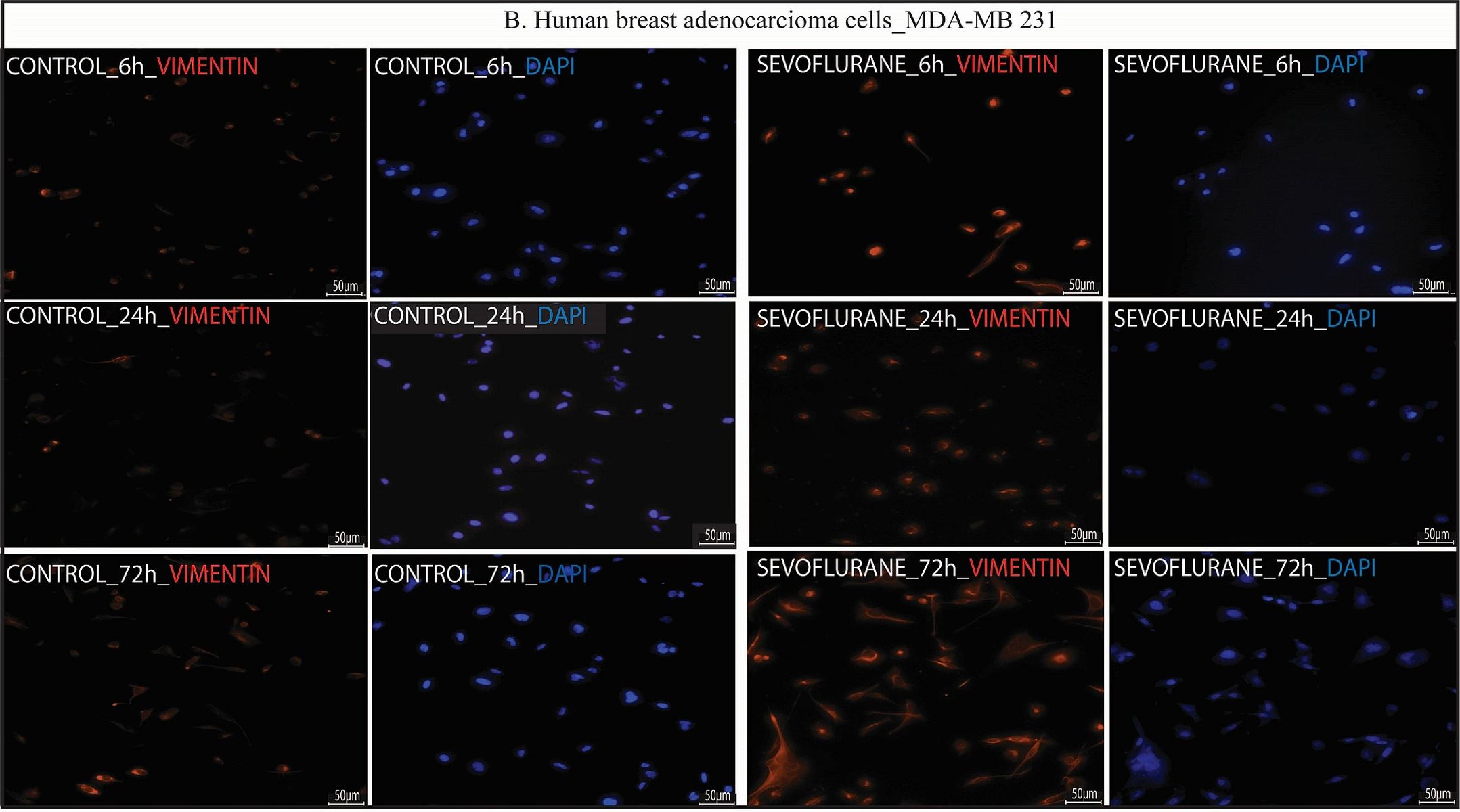

Supplement: Supplementary file 1 [file cimb-43-00022-s001.zip › SUPPLIMENTARY FIG. 5_B.jpg]
